# Early Airway Pathological Changes in Children: New Insights into the Natural History of Wheezing

**DOI:** 10.3390/jcm8081180

**Published:** 2019-08-07

**Authors:** Matteo Bonato, Mariaenrica Tiné, Erica Bazzan, Davide Biondini, Marina Saetta, Simonetta Baraldo

**Affiliations:** Department of Cardiac, Thoracic, Vascular Sciences and Public Health, University of Padova, 35128 Padova, Italy

**Keywords:** wheezing, bronchial biopsies, symptom persistence, clinical remission

## Abstract

Asthma is a heterogeneous condition characterized by reversible airflow limitation, with different phenotypes and clinical expressions. Although it is known that asthma is influenced by age, gender, genetic background, and environmental exposure, the natural history of the disease is still incompletely understood. Our current knowledge of the factors determining the evolution from wheezing in early childhood to persistent asthma later in life originates mainly from epidemiological studies. The underlying pathophysiological mechanisms are still poorly understood. The aim of this review is to converge epidemiological and pathological evidence early in the natural history of asthma to gain insight into the mechanisms of disease and their clinical expression.

## 1. Introduction

Asthma is a heterogeneous condition characterized by reversible airflow limitation with different phenotypes and clinical expressions [1,2]. Several studies on lung specimens from adult asthmatic patients have established that asthma is a process involving both the central and peripheral airways. The process includes chronic activation of the inflammatory response as well as structural changes of the airway wall—the latter is collectively called airway remodeling. The inflammatory response in asthma has been demonstrated to have a large heterogeneity and to involve both the innate (i.e., eosinophils, mast cells, innate lymphoid cells) and the acquired immunity (i.e., T-lymphocytes). Airway remodeling consists of shedding of the bronchial epithelium, thickening of the subepithelial reticular basement membrane (RBM) and of the smooth muscle mass, as well as proliferation of bronchial vessels or angiogenesis [3,4]. Although chronic inflammation in asthma is associated with airway remodeling, the mutual relation between the two is still a point of substantial debate. 

Although the histopathological features characteristic of asthma have been extensively described since the early 20th century, the molecular mechanisms responsible for the recruitment and activation of inflammatory cells and establishment of the architectural changes typical of airway remodeling are still only partially understood. The majority of studies in this field have considered adult and childhood asthma separately, almost as if they were different disease entities. At present, the evidence on asthma histopathology has been gathered exclusively from studies on adult cohorts, while there is a scarcity of studies on the histopathology of asthma in children. This is due essentially to the difficulty of obtaining bronchial biological samples from children and to the diagnostic dilemma—particularly in infants and preschool children—of discerning true asthma from other wheezing disorders. We suggest that a thorough comprehension of the histopathology of wheezing in children (i.e., at the beginning of the natural history of asthma) and of its interrelationship with the spectrum of clinical phenotypes should be the compass for guiding research into the jungle of inflammatory cells, mediators, and cytokines underlying asthma pathogenesis. Therefore, this review focuses on the major cellular and structural changes present in the airways of children with asthma in relation with the most important clinical phenotypes, in an attempt to integrate these data with those emerging from the longitudinal investigation of outcomes from early childhood to adulthood. 

## 2. Wheezing Disorders and Their Evolution across Developmental Ages

Wheezing is very common in the first 3 years of life. Yet, most preschool children with wheeze will be symptom-free by the time they reach school age, whereas only a minority will remain symptomatic, develop persistent wheeze, and ultimately be diagnosed as asthmatics. The identification of the factors which may predict the future development of asthma in early wheezing children has mainly been addressed by epidemiological studies [5], and is still a matter of vivid debate. 

While the great majority of wheezers do not usually progress to asthma in childhood and adolescence, some of them may have relapsing symptoms and be at increased risk of asthma in adulthood, and even of chronic obstructive pulmonary disease (COPD) later on [6]. Furthermore, the adult onset of asthma symptoms may occur in a considerable proportion of asthmatics, especially in women [7]. However, many patients do not completely remember their childhood symptoms, so it is unclear which cases might reflect relapse rather than true new onset.

Phenotypes with documented clinical importance in childhood asthma have been defined in several ways: (i) on the basis of concomitant traits, such as atopy; (ii) on the basis of the temporal pattern of symptoms appearance; (iii) on the basis of the longitudinal evolution of wheezing symptoms. As such, specific wheezing phenotypes that have been investigated in preschool children include: non-atopic and atopic wheezing; episodic and multiple-trigger wheezing; transient, persistent, and late-onset wheezing [2]. 

Prospective birth cohort studies such as the pioneering Tucson cohort have determined the longitudinal outcome of wheezing and defined specific categories, such as: (i) transient wheeze—children who wheeze during the first years of life, but not after the age of 3; (ii) persistent wheeze—children who start to wheeze before the age of 3 and maintain their symptoms even beyond school age; and (iii) late-onset wheeze—children who start to wheeze after the age of 3 [8]. However, these categories can only be recognized retrospectively, and are of little value in clinical practice [9].

The presence of atopy, and particularly an early sensitization to multiple allergens, is generally considered a fundamental risk factor for the future development of persistent asthma [8,10,11,12,13], although the relationship between wheezing and allergic sensitization in the first years of life is still controversial [14]. The predominant role of atopy was so generally accepted that often wheezing was considered by many pediatricians to truly represent asthma only when associated to atopy. Conversely, non-atopic wheezing was thought to be a transient phenotype, mostly triggered by viral infections [1,10,11,12,14,15,16]. On the same line, pediatricians proposed a clinical distinction between episodic and multiple-trigger (multitrigger) wheezing. Episodic wheezing is thought to be triggered by viral infections, manifests only in association with coryzal symptoms, and affected children are symptom-free between viral episodes, while multitrigger wheeze is triggered by multiple stimuli (including allergens, viruses, exercise, laughing) and is characterized by the presence of symptoms in between discrete episodes [2]. Multitrigger wheezing is usually considered as the phenotype associated with wheezing persistence over time [8,17]. However, some reports suggest that there is a wide overlap between the two phenotypes, with the wheezing pattern varying over time in many children [9], and that severe viral wheeze is equally associated with a high risk of asthma at school age [18]. 

Further important determinants of persistent symptoms include prenatal features (preterm birth and low birth weight), pulmonary function deficits and reduced breast feeding in early infancy, as well as indoor and outdoor exposures—particularly to environmental pollutants and cigarette smoking [19]. In the present review we focus mainly on the role of atopy and viral infections, the pathogenetic mechanisms of which are better known. 

## 3. Pathological Changes in Childhood Asthma

Endobronchial biopsy is the main diagnostic technique for evaluating the pathological processes in the bronchial mucosa directly [20,21]: it allows evaluation of the grade and type of inflammation, and provides evidence of the structural changes that may occur during the process of airway remodeling. 

Eosinophilic inflammatory infiltrate associated with thickening of the RBM, epithelial shedding, neo-angiogenesis, and smooth muscle enlargement are characteristic changes of asthma that have been widely described in adults [22,23], but scarcely investigated in children. 

**Figure 1:**
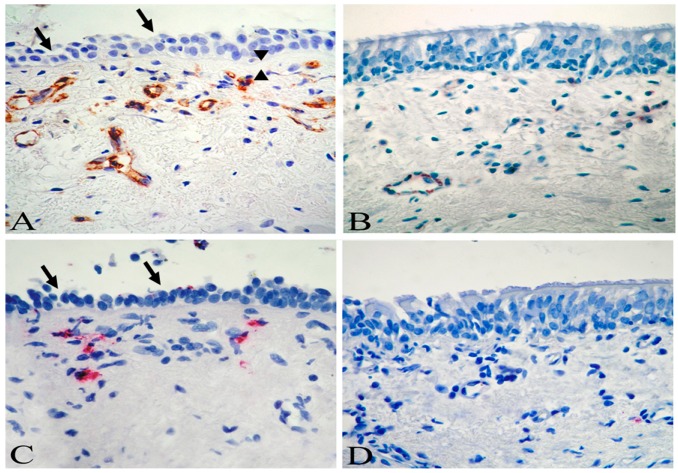
Biopsy sections from a child with asthma (**A**,**C**), and a control child (**B**,**D**). An increased number of subepithelial vessels (**A**, brown) and eosinophils (**C**, red) are demonstrated in the child with asthma. The arrows indicate loss of epithelial cells (**A**,**C**), while the arrowheads indicate reticular basement membrane thickening (**A**). Immunostaining with monoclonal antibody anti-CD31 (**A**,**B**) and anti-EG2 (**C**,**D**). Original magnification ×630. Reprinted with permission of the American Thoracic Society from [30].

The first pioneering study that investigated the bronchial histopathology in asthmatic children was by Cutz et al. in 1978, who described a prominent eosinophilic infiltrate and airway remodeling in the specimens of four asthmatic children [24]. After 20 years, Cokuğraş et al. [25] qualitatively examined bronchial biopsy specimens from 10 children with moderate asthma. They described a thickened RBM associated to an inflammatory infiltrate characterized by lymphocytic predominance, and only in one case they identified a prominent eosinophilic infiltrate. Subsequently, Jenkins and colleagues [26] reported a qualitative histopathological analysis of six children with severe asthma. They confirmed a thickened RBM and the presence of lymphocytes in lamina propria, but also recognized eosinophils in the inflammatory infiltrate and smooth muscle enlargement. These studies only performed qualitative analyses on a limited number of cases, and without a proper control group they can be considered as isolated observations. Payne and co-workers were the first to perform a quantitative evaluation of inflammatory and structural changes in a cohort of severe therapy-resistant asthmatic children. In two controlled studies [27,28], they demonstrated that asthmatic children had a thickened RBM in the absence of a prominent eosinophilia, suggesting that structural alterations can even precede inflammation in the natural history of the disease. However, the children examined in these two studies were severe asthmatics, most of them in treatment with maximal inhaled steroid therapy or even oral corticosteroids, which could have influenced the inflammatory process—particularly eosinophils. Conversely, our group has demonstrated in two consecutive studies that eosinophilic inflammation was definitely present in children with milder forms of the disease. Only a minority of children in our cohort were on inhaled corticosteroids (at low dose); the great majority of individuals were on as-needed salbutamol, and therefore free from the potential bias of steroid therapy [29,30]. Of importance, while earlier studies examined populations of school-aged children, our group was the first to assess histopathological changes typical of asthma in preschool children [30]. It is of interest that the early detection of airway eosinophilia in these children was associated with important features of airway remodeling—not only a thickened RBM, but also epithelial shedding and neo-angiogenesis (Figure 1) [30]. 

The presence of RBM thickening and eosinophilic inflammation was subsequently confirmed in an even younger cohort of children [31] (median age 2.4 years), and was further investigated in several cohorts whose results are summarized in Table 1 [32,33,34,35,36,37,38]. Taken together, these studies showed that most of the structural and inflammatory changes typical of asthma are present in asthmatic children, and also in preschool wheezing children, that is, at the beginning of the natural history of the disease. In particular, almost all reports converged on showing a thickened basement membrane, indicating that this is an early event present from 2–3 years of life. Whether RBM thickening is already present in younger infants (1 year of age) is actually a matter of debate, since it was not found in a cohort of 16 infants by Saglani et al. [36], but has been recently reported in a larger cohort of 30 infants by Berankova and coworkers [34]. Some incongruities regarding eosinophilic airway inflammation have been reported, but these could reflect the different treatment levels in different cohorts, rather than true pathogenetic differences as also suggested by other authors [39]. Finally, other aspects of airway remodeling (epithelial loss, angiogenesis, and smooth muscle enlargement have been examined in limited reports [30,33,37,38], and deserve further investigation. 

In conclusion, these studies conducted in the last twenty years, despite their unquestionable limitations (small cohorts, influence of steroid treatment, and diagnostic wheezing dilemma in preschool children) provide evidence that both inflammation and remodeling are present early in the natural history of the disease, challenging the “classic theory” of asthma pathogenesis which views remodeling as a consequence of a long-lasting chronic inflammation. These observations support the hypothesis that the epithelial–mesenchymal signaling may play a fundamental role in the development of bronchial asthma and its clinical phenotypes. In fact, the chronic damage to the airway epithelium due to a variety of stimuli could activate inflammatory pathways, with the release of damage-related cytokines—especially IL-33, which is hyperexpressed in children and directly correlated to RBM thickness [40], but also IL-25, TSLP, and mitotic/fibrogenic growth factors, thereby promoting angiogenesis as well as thickening of the RBM and smooth muscle [41,42,43]. The stimuli which have been more extensively investigated are allergens and viral pathogens, and we will now review the evidence in the literature on the activation of these pathogenetic mechanisms in relation to airway histopathological changes in childhood asthma. 

## 4. Atopy and Related Pathological Changes

Atopy is generally considered to be a crucial feature characterizing asthma either in children or in adults, and early onset allergic asthma is considered to be the archetypal phenotype of the disease. 

The term atopy (from the Greek “atopos”, meaning “out of place”) describes the tendency to be hyperallergic, the genetic propensity to mount an IgE response to triggers including pollens, animal dander, and food-based allergens. Allergens are well known triggers of type 2 immunity characterized by the differentiation of naïve T CD4^+^ cells towards Th2 effector cells, which is typically associated with IgE production, eosinophilia, and mast cell activation. The keystone cytokines in type 2 immune response include IL-4, IL-5, IL-9, and IL-13. IL-4 is crucial for the differentiation of naïve Th0 cells to Th2 cells, which in turn induces isotype switching to IgE production. Specific IgE antibodies bind to their high-affinity FceRI receptors on the surface of basophils or mast cells, leading to the sensitization of those cells. IL-5 and IL-9 are responsible for the activation and recruitment of eosinophils and mast cells, respectively, while IL-13 induces goblet cell hyperplasia, mucus hyper-secretion, and airway hyper-responsiveness [44].

Atopic sensitization, with the resulting activation of the Th2 cascade, has long been considered a key determinant of wheezing persistence and asthma development in childhood. Indeed, it has been reported in several cohorts that children who have either a family history of allergies or who will become sensitized to local aeroallergens are more likely to have wheezing that persists into adulthood, whereas wheezing appears to resolve in adolescence in those children who do not develop atopic sensitization [15]. Based on this evidence, we investigated the hypothesis that a different airway pathology could be present in atopic and non-atopic wheezing children. In a well-characterized cohort of children in whom symptoms of wheezing were those typical of asthma (multitrigger, responsive to bronchodilators), we reported that all the histopathological traits of asthma were observed in both atopic and non-atopic children [45]. These traits included RBM thickening, epithelial desquamation, angiogenesis, and even an eosinophilic inflammatory infiltrate with upregulation of Th2 cytokines IL-4 and IL-5. Further work from our group demonstrated that atopic and non-atopic wheezing children have a similar degree of eosinophilic inflammation even in bronchoalveolar lavage (i.e., eosinophils and eosinophil cationic protein levels) [46]. These data in children complemented the seminal observations by Humbert and co-workers, who showed more similarities than differences in the immunopathology of atopic and non-atopic asthma in adults [47]. In conclusion, studies on bronchial biopsies and BAL demonstrated the similar nature of the histopathological substrate of these two crucial wheezing phenotypes from the beginning of the disease. 

## 5. Viral Infections and Related Pathological Changes

Rhinovirus infections are among the most frequent cause of asthma exacerbations in adults, but even more so in children [48,49,50]. Indeed, cold-related wheezing is the most common respiratory symptom in preschool children, with up to 40%–50% of children experiencing at least one wheezing episode before the age of three. However, recurrent wheeze in early childhood is not always asthma. Recent techniques with the molecular detection of viral pathogens brought significant advances to understand the relationship between viral infections and asthma inception. Not only viruses are frequently isolated in exacerbations of asthma, but respiratory viral infections in early life—particularly rhinovirus and respiratory syncytial virus (RSV)—are associated with increased risk of asthma later in life [18,51,52,53,54,55,56]. 

Defective production of type I and type III interferons (IFNs) upon rhinovirus infection has been documented in adults with asthma, which can lead to impaired viral clearance, thus aggravating the impact of infections on the lung [57,58]. We demonstrated that such impaired immune response by epithelial cells was already present in preschool children with asthma [59]. Of importance, we found that impaired innate lung immunity was associated with structural changes in the airway biopsies (epithelial loss) and to markers of type 2 immunity [59]. We then investigated whether such deranged antiviral response can be considered as a risk for future asthma persistence. After an 8-year follow-up, we showed that children with asthma persisting at adolescence already had deficient IFN production and higher viral replication at preschool age [60]. These findings suggested the hypothesis that the immunologic interactions between viral infections and type 2 immunity predisposes to more severe acute responses to the virus, resulting in chronic insults to the epithelium, and eventually leading to the development of asthma. These observations are also in agreement with findings from the follow-up of large cohorts of children in the Tucson and the COPSAC studies, where children with asthma at school age already exhibited aberrant immune responses in infancy, not only to viruses but also to bacteria [61,62]. Altogether, these observations would support the concept that an aberrant response to infectious pathogens very early in life—mainly driven by the airway epithelium—is a crucial determinant of the evolution toward asthma.

## 6. Evolution of Asthma Symptoms in Relation to Pathological Changes

**Figure 2:**
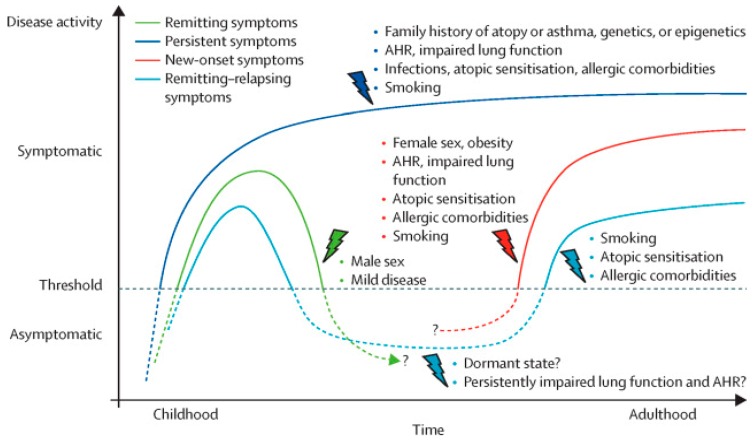
Determinants of disease course across asthma transition and ages. The figure displays putative determinants that affect the disease course of different asthma phenotypes by course and time of onset of symptoms. AHR: airway hyper-responsiveness. Reprinted with permission from Elsevier from [5].

The last decades have seen a rapid growth of information on the evolution of asthma-like symptoms in childhood and their determinants. Most infants and young children with wheezing will outgrow their symptoms as their lungs develop, but some will persist in their symptoms over time toward confirmed asthma (Figure 2) [5,63]. Those transient wheezers who do not usually progress to asthma in childhood and adolescence can still have symptoms remittance in adulthood, and can be at increased risk of COPD [6]. Emerging evidence from clinical and epidemiological studies that followed-up children from the care of their pediatrician into adolescence and then into adulthood has helped us to understand the main determinants of symptoms persistence [5]. Early aeroallergen sensitization, respiratory infections in infancy, and cigarette smoking exposure have all been associated with persistent symptoms [19]. Reduced lung function in early infancy (as soon as 1 month of age) is another factor that has been associated with persistent wheezing at 11 years [3]. This has been confirmed by the results of a number of studies [4,10,11,14,15], which have demonstrated that poor airway function shortly after birth (at 2–3 months of age) is a risk factor for asthma in teenagers and young adults (at 11, 16, and 22 years of age). This effect may reflect the variation in genes regulating normal lung growth and airway structure during lung development [64,65]. Furthermore, the association between lung function and symptoms persistence is independent of the effect of airway hyperresponsiveness, atopy, and type 2 allergic inflammation (as measured by blood eosinophil levels) [66]. These findings indirectly suggest that airway structural changes, which start early in life, are crucial determinants of the persistence of asthma symptoms and therefore highlight the need for a better understanding of the pathogenetic mechanisms underlining symptom persistence.

To our knowledge, only four longitudinal studies have investigated the pathological changes able to predict the presence of asthma at follow-up. The first two, by Malmström and co-workers, evaluated a cohort of 53 infants with pathological changes and airway conductance measured at baseline and then re-evaluated them at 3 and 8 years of age. While there was a correlation between RBM thickening and mucosal mast cells with corticosteroid purchase at 3 years (as an indirect index of asthma) [67], the correlations were not present when children were comprehensively reassessed for asthma at 8 years of age [68]. Indeed, no pathologic features at baseline correlated with the presence of confirmed asthma at school age [68]. Then, O’Reilly and co-workers reported a follow-up of a cohort of 47 preschool children with severe recurrent wheezing [69]. RBM thickening and the eosinophilic infiltrate, despite being distinctive features of wheezing children at baseline, were not able to distinguish children who did or did not develop asthma at follow-up. Conversely, airway smooth muscle mass, which was not enlarged at baseline in symptomatic children, was the only histological feature to be associated to the development of asthma at school age. More recently, our group has also completed a clinical follow-up of a cohort of 80 preschool wheezing children (and non-wheezing controls) that had histological parameters assessed at baseline. At variance with previous studies, a thicker RBM and an eosinophilic inflammation in the lamina propria were clearly associated to the persistence of asthma from preschool to school age. When we performed a multivariate analysis, only RBM thickening remained a significant predictor of asthma persistence. Even when we limited our analysis to toddlers only (children under 3 years), RBM thickening at this early age remained a significant predictor of asthma later in life [70]. 

While the persistence of wheezing from early life to school age is associated with abnormal histological traits, it is also important to know what happens to these traits with the remission of symptoms. Marshall and co-workers compared the lung function and sputum cellularity of children with persistent or transient wheezing after following them through adolescence [71]. They found that airway eosinophilic inflammation and lung function impairment were seen not only in persistent wheezers, but also in children with a transient wheezing phenotype. Similarly, Dutch cohorts that examined young adults in clinical remission from childhood asthma reported persistent airway remodeling despite symptoms disappearance [72,73], suggesting that symptoms remission does not equate remission of the underlying pathology. In support of this hypothesis is the observation that, when assessed with functional tests, the majority of patients in apparent clinical remission still retain impaired lung function and airway hyperresponsiveness [74]. 

This topic now becomes truly fascinating, since pathological changes in children that predispose them to symptoms persistence are also present in subjects in clinical remission during adolescence/early adulthood. To disentangle this issue, we have created a number of different wheezing phenotypes (atopic/non-atopic, multitrigger/episodic, transient/persistent/late onset, etc.). However, there is no doubt that we are forcing an extensive variety of clinical phenotypes into artificially simple categories—all of them unable to fully capture the real complexity of a disease that is variable by definition. Instead of being distinct pathogenetic entities, these wheezing phenotypes may rather represent different clinical expressions of the same underlying pathology (i.e., different levels of disease severity). Let us imagine the process as a continuum: wheezing children develop airway structural changes very early in life and keep all the pathological hallmarks of asthma also in adolescence, and maybe for their entire life, independently from the clinical “activity” of the disease. To elicit symptoms, a certain threshold of trigger is needed at a given time point; with lower burdens, individuals will remain asymptomatic and this will occur mostly during adolescence/early adulthood when they achieve their maximal levels of pulmonary function. Nonetheless, these subjects, even if in clinical remission, have all the pathological hallmarks of asthma in their airways and will be prone to relapse whenever exposed to a high burden of inflammatory stimuli. The most harmful factor in adolescence is cigarette smoking, against which they should be strongly advised. With the recent widespread use of e-cigarettes in adolescents, whose effects on the lungs are a cause of strong concern, we also need to look carefully at the possible effects of e-cigarette use in these vulnerable children. 

The description of the clinical course of asthma and pulmonary function trajectories during the entire human life in relation to airway histopathology is crucial for matching the wide spectrum of asthma phenotypes—from early wheezing in infancy to the late-onset adult forms—with specific pathological traits. We have reviewed here the available data focusing on the early stages of the disease to address the pathology at the onset of asthma symptoms and then investigated its relation with the evolution of symptoms during childhood and adolescence. With the upcoming follow-up of existing cohorts across the whole natural history of asthma, further into adulthood and elder age, we will have a unique opportunity to unravel the mechanisms behind this multifaceted disease. 

## Figures and Tables

**Table 1:** Histopathologic changes in bronchial biopsies of asthmatic/wheezing children.

	Number of Children	Mean Age (years)	RBM Thickening	Epithelial Loss	SM Enlargement	Submucosal Inflammation	Angiogenesis
Cutz 1978 [24]	4	12 (11–12)	+	0	+	Eos	/
Cokuğraş 2001 [25]	10	9.3 ± 3.8	+	/	/	Ly	/
Jenkins 2003 [26]	6	13.5 (6–17)	+	/	+	Eos Ly	/
Payne 2003 [27]	19	13 (6–16)	+	/	/	0	/
Barbato 2003 [29]	9	8 (4–12)	+	/	/	Eos	/
Payne 2004 [28]	36	13 (6–16)	+	/	/	0	/
Saglani 2005 [36]	16	1 (0.3–2)	0	/	/	0	/
Barbato 2006 [30]	17	5 (2–15)	+	+	/	Eos	+
Saglani 2007 [31]	16	2.4 (0.6–4.75)	+	/	/	Eos	/
Kim 2007 [32]	18	13 ± 1	+	/	/	/	/
Regamey 2008 [37]	24	12.5 (6.7–15.8)	/	/	+	/	/
Zhou 2011 [33]	13	7.2 (1.5–15)	+	+	+	Ly Eos	/
Bossley 2012 [38]	53	12 (9–14)	+	/	+	Eos	/
Berankova 2014 [34]	30	1 (0.3–3.3)	+	/	/	/	/
Van Mastrigt 2015 [35]	107	9.5 ± 4.6	+	/	/	/	/

Age is reported as median (range) or mean ± SD. Definition of abbreviations: Eos: eosinophils; Ly: lymphocytes; RBM: reticular basement membrane; SM: smooth muscle. Presence of the histological feature (+), absence (0), feature not evaluated in the study (/).
